# CSCVAE-NID: A Conditionally Symmetric Two-Stage CVAE Framework with Cost-Sensitive Learning for Imbalanced Network Intrusion Detection

**DOI:** 10.3390/e27111086

**Published:** 2025-10-22

**Authors:** Zhenyu Wang, Xuejun Yu

**Affiliations:** College of Computer Science, Beijing University of Technology, Beijing 100124, China; jmxwzy0510@emails.bjut.edu.cn

**Keywords:** network intrusion detection, anomaly detection, class imbalance, conditional variational autoencoder, cost-sensitive learning

## Abstract

With the increasing complexity and diversity of network threats, developing high-performance Network Intrusion Detection Systems (NIDSs) has become a critical challenge. A primary obstacle in this domain is the pervasive issue of class imbalance, where the scarcity of minority attack samples and the varying costs of misclassification severely limit the effectiveness of traditional models, often leading to a difficult trade-off between high False Positive Rates (FPRs) and low Recall. To address this challenge, this paper proposes a novel, conditionally symmetric two-stage framework, termed CSCVAE-NID (Conditionally Symmetric Two-Stage CVAE for Network Intrusion Detection). The framework operates in two synergistic stages: Firstly, a Data Augmentation Conditional Variational Autoencoder (DA-CVAE) is introduced to tackle the data imbalance problem at the data level. By conditioning on attack categories, the DA-CVAE generates high-quality and diverse synthetic samples for underrepresented classes, providing a more balanced training dataset. Secondly, the core of our framework, a Cost-Sensitive Multi-Class Classification CVAE (CSMC-CVAE), is proposed. This model innovatively reframes the classification task as a probabilistic distribution matching problem and integrates a cost-sensitive learning strategy at the algorithm level. By incorporating a predefined cost matrix into its loss function, the CSMC-CVAE is compelled to prioritize the correct classification of high-cost, minority attack classes. Comprehensive experiments conducted on the public CICIDS-2017 and UNSW-NB15 datasets demonstrate the superiority of the proposed CSCVAE-NID framework. Compared to several state-of-the-art methods, our approach achieves exceptional performance in both binary and multi-class classification tasks. Notably, the DA-CVAE module is designed to be independent and extensible, allowing the effective data that it generates to support any advanced intrusion detection methodology.

## 1. Introduction

With the rapid development of big data, cloud computing, and high-speed communication technologies, global networks have penetrated all aspects of social life with unprecedented depth and breadth. The dependence of modern society on cyberspace has grown to an unparalleled degree, underlying everything from the stable operation of critical infrastructure and corporate digital transformation to personal communication and entertainment [1,2,3]. The network, acting as the central nexus for information exchange and service interaction, concurrently presents a significant attack surface due to its intrinsic openness and complexity, making it a prime target for a wide range of malicious activities and cyber threats [4,5]. Consequently, developing a proactive, efficient, and precise cybersecurity architecture to safeguard the stability and security of cyberspace remains a perennial and central challenge for the networking technology community. Among the numerous defense technologies, Network Intrusion Detection Systems (NIDSs) play a crucial role. They work by continuously monitoring and analyzing network traffic to detect and issue alerts for potential attack behaviors and security threats in real time. Recent years have witnessed the advent of numerous innovative intrusion detection techniques, developed in response to the increasing complexity of cyber threats [6,7,8]. The intrusion detection task is commonly framed in academia and industry as a binary classification problem (normal vs. anomalous) or a multi-class classification problem (identifying specific attack types). Based on this foundation, a variety of classic machine learning algorithms have seen extensive application in this field, including decision trees, support vector machines, multilayer perceptrons, and random forests [9,10]. Ref. [9] introduced the Dendron framework, which employs a combination of decision trees and genetic algorithms to evolutionarily generate highly readable intrusion detection rules. The framework also incorporates heuristics targeting minority classes, thereby improving the detection of rare attacks. Ref. [10] utilized classic classifiers, such as random forests, Bayesian networks, LDA, and QDA, to conduct binary and multi-class classification experiments on the CICIDS2017 dataset [11]. Their work effectively addressed the bottleneck related to both classification performance and computational efficiency when dealing with high-dimensional network traffic data. Nevertheless, the efficacy of these traditional methods is constrained by their inherently shallow architecture when confronted with the massive, high-dimensional, and intricate nature of modern network traffic data, limiting their capacity for effective feature learning and representation. Deep learning approaches, in contrast, exhibit superior performance in processing high-dimensional, complex, and noisy data. This superiority stems from their inherent ability to perform powerful non-linear transformations and automatically extract hierarchical features. As a result, the integration of deep learning technologies into network intrusion detection has become a highly active area of research. Convolutional Neural Networks (CNNs) [12], Recurrent Neural Networks (RNNs) [13], and autoencoders (AEs) [14], in particular, stand out as the most prevalent and representative models currently employed in this domain. Ref. [12] proposed a tree-like CNN architecture that leverages a hierarchical structure in conjunction with the Soft-Root-Sign (SRS) activation function. This design facilitates accelerated training and has demonstrated superior detection accuracy for specific attack classes, notably DDoS, infiltration, and brute-force attacks. Ref. [14] introduced a two-stage learning approach, integrating a Conditional Variational Autoencoder (CVAE) with Extreme Value Theory (EVT), which substantially enhanced the model’s ability to detect unknown attacks. Nevertheless, while many advanced intrusion detection methods have been proposed, several pressing challenges persist in practical applications. Firstly, acquiring effective attack samples is a process that is both resource-intensive and prohibitively expensive. The process of collecting and annotating diverse attack traffic from live network environments is often a labor-intensive and computationally expensive endeavor. This intrinsic challenge in sample collection is a direct cause for the severe class imbalance prevalent in most public datasets, which are characterized by a profound scarcity of attack samples relative to normal ones. Secondly, existing methods exhibit limited performance when processing datasets characterized by severe class imbalance. In the context of attack categories characterized by extreme data sparsity, neither traditional techniques for addressing class imbalance (such as SMOTE [15]) nor standard deep learning classifiers can reliably learn robust, generalizable discriminative features. This inherent limitation ultimately results in a classification performance that is often unsatisfactory. To overcome the aforementioned limitations, this paper introduces a novel two-stage framework for network intrusion detection, termed CSCVAE-NID. Specifically, our framework first employs a DA-CVAE network for data augmentation, which leverages a parallel dual-encoder structure to disentangle the content features of anomaly samples from the style features of their attack categories. This design effectively mitigates the class imbalance issue arising from the insufficient number of minority-class attack samples in the training data. For the second stage, aiming for high-precision multi-class classification, we designed the CSMC-CVAE network by symmetrically swapping the inputs of the parallel dual-encoder structure and slightly altering the decoder architecture. This novel approach reframes the classification task as an inference process based on probabilistic distribution matching, which involves measuring the consistency between the content feature distribution of a given sample and the prototypical distribution of each potential class. Moreover, to augment the training robustness of the CSMC-CVAE in class-imbalanced scenarios, a cost-sensitive learning strategy was integrated. This was implemented by introducing a predefined cost matrix into the loss function, where each element quantifies the penalty for a specific type of misclassification. This approach ensures that the model’s optimization process is guided not just by classification accuracy but also by the real-world costs of prediction errors. Comprehensive experiments on the public CICIDS2017 [11] and UNSW-NB15 datasets [16,17,18] demonstrate that our proposed method significantly outperforms existing state-of-the-art approaches in both binary and multi-class classification tasks. The specific contributions of our proposed method can be summarized as follows:

1. We propose a data augmentation scheme based on a Conditional Variational Autoencoder (CVAE), namely, the DA-CVAE model. This model leverages the attack category as a condition to directionally generate high-quality and diverse synthetic samples for minority classes within the dataset. Consequently, it effectively counteracts the training bias induced by the severe class imbalance, a common issue in such data.
2. To enable high-precision multi-class classification, we developed the CSMC-CVAE model, which operates on the fundamental principle of matching probabilistic distributions. This model reframes the classification task as a problem of quantifying the consistency between a given sample’s feature distribution and the prototypical distribution of each candidate class.
3. To further address the issue of class imbalance at the algorithmic level, we incorporated a cost-sensitive learning strategy within the CSMC-CVAE’s training paradigm. This was achieved by augmenting the loss function with a predefined cost matrix, which effectively forces the optimization process to prioritize the correct classification of samples from underrepresented classes.
4. Experimental results on two public datasets indicate that our proposed CSCVAE-NID framework significantly outperforms both conventional and state-of-the-art methods in terms of detection accuracy.

The rest of this paper is structured as follows: In Section 2, we review the relevant literature. Section 3 provides a detailed description of our proposed CSCVAE-NID methodology. Section 4 presents the comprehensive experimental validation of CSCVAE-NID’s performance on public benchmark datasets. Finally, Section 5 concludes the paper and discusses future work.

## 2. Related Work

### 2.1. Machine Learning-Based Network Intrusion Detection

The proliferation of inter-user communication has led to a sustained increase in anomalous network traffic, thereby introducing substantial security risks. In an effort to effectively curtail the disruption caused by anomalous intrusions to inter-device communication, a portion of researchers have dedicated their work to the development of machine learning-based network intrusion detection methods [19].

These methods often necessitate manual statistical feature engineering. In the study by Dogan [20], a set of features including flow duration, packet length, arrival time, and time intervals was selected. By applying tree-based classification algorithms to these features, a detection rate of 90% was attained. In their work [21], Lawa et al. surveyed and compared various anomaly detection mechanisms within the networking domain. They also benchmarked the performance of several anomaly classification methods on the UNSW-NB15 dataset. In an effort to bolster the performance of machine learning techniques, ref. [22] devised a two-stage classification framework that synergistically combines machine learning with Deep Packet Inspection (DPI). The primary objective of this architecture is to capitalize on the computational efficiency of machine learning while harnessing the high recognition accuracy afforded by DPI. However, this method is unable to handle encrypted traffic, as Deep Packet Inspection (DPI) necessitates access to the raw payload content. Furthermore, the computationally intensive nature of DPI restricts its applicability in real-time settings. The work presented in [23] introduces an ensemble classification technique grounded in statistical flow features. Evaluation on the UNSW-NB15 dataset revealed that this model achieves a high level of detection accuracy while maintaining a low false positive risk. Ref. [24] presents a thorough investigation into the relative efficacy of single machine learning classifiers as opposed to ensemble strategies for detecting anomalous network behaviors. Nevertheless, a more thorough evaluation of the computational cost and real-time performance of these models is still required. Furthermore, the significant time investment demanded by manual feature engineering continues to be a major challenge for machine learning-based network intrusion detection approaches.

### 2.2. Deep Learning-Based Network Intrusion Detection

Whereas traditional machine learning relies on manual feature engineering, the primary advantage of deep learning is its capability for automatic feature extraction. Ref. [25] employed Long Short-Term Memory (LSTM) to classify the ISCX VPN-NonVPN traffic and also discussed the applicability of Convolutional Neural Networks (CNNs) for processing this type of traffic. Their experiments achieved an accuracy of 91% on the dataset. Ref. [26] introduced a lightweight CNN (LNN) for classifying the BoT-IoT dataset, attaining an accuracy of 96.15%. Despite its advantages over conventional CNNs in model complexity and computational efficiency, the robustness of its feature extraction capabilities was not demonstrably improved.

To improve multi-class classification of anomalous traffic, deep learning approaches frequently utilize ensemble strategies involving multiple neural networks. As an illustration, Ref. [27] devised a hybrid architecture for traffic identification and classification on the BoT-IoT dataset by integrating Long Short-Term Memory (LSTM) with autoencoder (AE) networks. The performance of this model in multi-class classification tasks was thereby substantially elevated. A limitation of this work, however, is the narrow scope of its comparative study. The investigation was confined to exploring performance variations among different optimizers and CNN architectures, omitting a direct, horizontal comparison with other classes of deep learning models. A hybrid neural network model for anomaly detection grounded in traffic features was proposed in [28]. The architecture assigns distinct roles to its components: a Convolutional Neural Network (CNN) is employed for the extraction of sequential features, whereas a Deep Neural Network (DNN) is specialized for learning high-dimensional feature representations. A key advantage of this model over other deep learning methods is its ability to concurrently achieve high classification accuracy while maintaining a lower model complexity. An unsupervised and lightweight anomaly detection model, ARCADE, was proposed in [29]. The model synergistically combines a one-dimensional convolutional autoencoder (1D-CNN AE) with a Wasserstein GAN with Gradient Penalty (WGAN-GP) adversarial regularization strategy. Notably, it operates directly on raw traffic bytes to perform unsupervised anomaly detection. Nevertheless, the model exhibits subpar performance in detecting flood attacks, achieving F1-scores of only 68.70% and 66.61% for these two categories, respectively. Ref. [30] presents a multi-factor feature-encoding methodology grounded in autoencoders. It formulates a geometry-aware feature representation through the joint consideration of latent representations, reconstruction residual directions, and reconstruction errors. A key component of this approach is a specialized network engineered to inject reconstruction error information into the representation. The method demonstrates a substantial performance enhancement in weakly supervised anomaly detection, especially in scenarios with limited labeled data. Nevertheless, it is constrained by two factors—a relatively high computational complexity, and a dependency on data manifold assumptions—which could potentially hinder the capture of complex anomaly patterns.

In summary, while existing deep learning methods demonstrate considerable potential for intrusion detection, they are still beset by several prevalent limitations. On the one hand, many advanced models feature increasingly complex network architectures, which incorporate multiple neural networks or intricate feature-encoding modules. This complexity results in significant computational overheads and challenges in practical deployment. On the other hand, existing research has generally not sufficiently considered the negative impact of data imbalance on multi-class detection performance. Most models are still designed with the primary goal of improving overall accuracy, while paying insufficient attention to the recognition capability for minority attack classes and the risk costs associated with different misclassification types. This ultimately limits their reliability in real-world network environments.

### 2.3. Imbalanced Data Handling in Network Intrusion Detection

A fundamental challenge in network intrusion detection, when treated as a classification task, is the pervasive issue of imbalanced class distributions [31,32]. This is often manifested by a stark disparity in sample sizes, where some classes comprise tens of thousands of instances while others are represented by only several hundred. This class imbalance severely undermines the generalization performance of a model, with the detection of minority classes being the most adversely affected.

Generally, the approaches to mitigate class imbalance in network intrusion detection are primarily bifurcated into two categories: data-level strategies and algorithm-level strategies.

Data-level strategies [33,34,35] aim to balance the dataset prior to classification training by modifying the sample distribution via oversampling or undersampling techniques. Oversampling [36] aims to augment the minority class by generating additional samples, whereas undersampling seeks to reduce the majority class by removing existing samples. For instance, Tan et al. [37] developed an effective anomaly detection model by integrating the SMOTE oversampling technique with a random forest ensemble. In order to enhance intrusion detection performance, Wang and Septian [38] devised a multi-stage detection framework that incorporates both data sampling techniques and deep learning models. The framework operates in three key stages: initially, selecting the most informative features via an ensemble of decision trees; subsequently, balancing the dataset by augmenting the minority class with the SMOTE algorithm; and ultimately, feeding the processed data into a Recurrent Neural Network (RNN) for final attack identification. The study by Al and Dener [39] illustrates the synergy between hybrid sampling at the data level and a hybrid architecture at the model level. Their data balancing process involves a two-step hybrid approach. First, the SMOTE algorithm is employed to oversample the minority class. This is followed by an undersampling step, where the Tomek links method is used to eliminate noisy or borderline instances from the majority class. Building upon this, they constructed a deep model combining a CNN with LSTM, where the CNN extracts spatial features and the LSTM captures temporal dependencies, ultimately performing the intrusion detection task.

From the perspective of model learning, both predominant sampling techniques present potential issues. By discarding samples from the majority class, undersampling risks causing the model to learn an incomplete representation of that class, potentially introducing information bias. Conversely, oversampling techniques, by augmenting the dataset with synthetic samples, compel the model to learn a decision boundary for a more complex, artificially expanded data distribution. This inherently increases the risk of the model overfitting to the training data.

A distinct paradigm for addressing class imbalance involves modifications at the algorithm level [40,41,42,43]. In contrast to data-level strategies, this approach does not manipulate the data distribution. Instead, it focuses on redesigning or adapting the learning algorithm itself, such that it can effectively learn from an imbalanced training set without bias. A quintessential example of an algorithm-level strategy is cost-sensitive learning [44]. This methodology confronts class imbalance by modifying the optimization objective, i.e., the loss function. Whereas standard models assign a uniform cost to all classification errors, cost-sensitive learning introduces a predefined cost matrix. This matrix is designed to heavily penalize the misclassification of minority class instances as members of the majority class, thereby forcing the model to pay greater attention to them. As a result, during the iterative parameter optimization, the model is compelled to preferentially reduce classification errors on the minority class. This ultimately yields a higher degree of recognition accuracy for the underrepresented category. The work of Telikani and Gandomi [45] serves as a prime example of the application of cost-sensitive learning. They put forward a deep learning model, termed the Cost-Sensitive Stacked Autoencoder (CSSAE), tailored specifically to the challenge of data imbalance in anomaly detection. The fundamental principle of this model is the establishment of a cost matrix to assign disparate penalty weights to different types of misclassification errors, thereby compelling the model to learn more effectively from the imbalanced data.

Motivated by these pioneering works, the synergistic combination of cost-sensitive mechanisms and various Deep Neural Network architectures has become a prominent and active research avenue. Researchers have incorporated asymmetric costs into various deep learning models, including autoencoders, Convolutional Neural Networks (CNNs), and Recurrent Neural Networks (RNNs). The objective of this integration is to harness the potent feature extraction capabilities of deep learning, while concurrently mitigating learning biases on imbalanced data via cost-sensitive strategies. This approach ultimately leads to enhanced performance in complex detection tasks. Ref. [45] provides a tangible example of cost-sensitive deep learning in practice. The contribution is twofold: Firstly, the authors devised a methodology for constructing the cost matrix wherein the costs are inversely proportional to the class sample counts. Secondly, these derived cost weights are directly incorporated into the loss function, thereby compelling the model to improve its recognition capabilities for minority classes. Despite achieving an approximate 2% improvement in accuracy on the VPN-NonVPN dataset, the cost integration mechanism of this method presents a theoretical deficiency. The primary issue is that the model’s output vector may forfeit its characteristic as a standard probability distribution, meaning that the summation of class probabilities is not guaranteed to equal one.

This study aims to overcome the limitations of existing methods by positioning the cost-sensitive learning strategy as the central component of the algorithm-level imbalance handling within our CSCVAE-NID framework. Drawing upon this concept, we also employ a weighting scheme where the cost for each class is set to be inversely proportional to its sample frequency. These cost weights are incorporated into a weighted cross-entropy loss formulation, which, in turn, forms an integral part of the joint loss function for our CSMC-CVAE sub-framework. This dual approach allows us to leverage the cost-sensitive strategy to force the model to prioritize the correct classification of minority attacks during training, while simultaneously enabling risk-aware decisions via our unique CVAE classification mechanism based on matching sample content distributions with prototypical class distributions.

## 3. The Proposed Methodology

### 3.1. Overview

**Figure 1:**
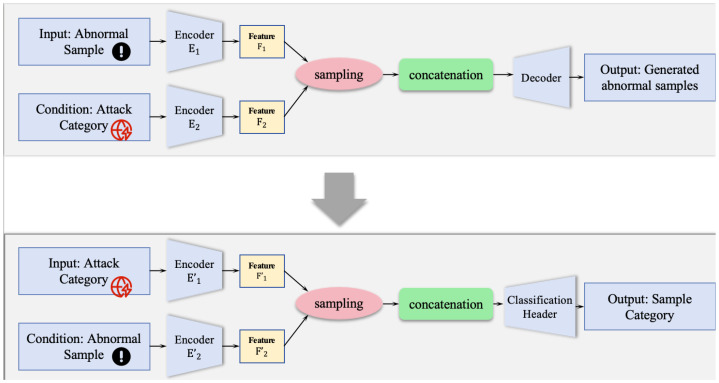
Overview of the proposed CSCVAE-NID framework. Blue boxes represent inputs/outputs, light blue trapezoids are encoder/decoder modules, yellow boxes are features, the pink oval is the sampling process, and the green box is the concatenation operation.

As depicted in Figure 1, the proposed CSCVAE-NID model comprises two sub-frameworks. The first of these, the DA-CVAE, is a data augmentation module that takes the specific attack category as a condition to generate designated anomaly samples, thus expanding the sample size for minority attack classes. The second sub-framework, which we term the CSMC-CVAE, utilizes the anomaly sample as a condition to execute the multi-class network intrusion classification task. A key feature of this sub-framework is the integration of a cost-sensitive learning strategy. This strategy assigns differential classification costs—manifested as weights in the loss function—to various classes, thereby compelling the model to prioritize the correct classification of high-cost categories during training. In the following subsections, we will detail the specific architectures of the two CVAEs, as well as the implementation of our cost-sensitive learning strategy.

### 3.2. DA-CVAE for Data Augmentation

Class imbalance represents a prevalent and formidable challenge in real-world network intrusion detection tasks. Not only does the number of normal samples vastly outnumber attack samples, but there is also a substantial imbalance in the sample distribution across different attack categories. This form of data skewness induces a bias in the classification model during training, causing it to disproportionately focus on the majority class. As a consequence, the model often fails to adequately learn the discriminative patterns of minority attacks, which ultimately leads to a degradation in detection performance for these rare events. To alleviate this issue, we first employ a Conditional Variational Autoencoder dedicated to data augmentation, designated as the DA-CVAE (Data Augmentation Conditional Variational Autoencoder).

As depicted in the upper part of Figure 1, the primary goal of this module is to learn and model the data distribution of minority attack classes. It focuses on anomaly samples from these underrepresented categories, generating high-quality and diverse synthetic samples conditioned on the given class label.

To achieve a more effective disentanglement of the sample-specific content information and the class-specific style information, we engineered a parallel dual-encoder architecture. This architecture is composed of two parallel sub-networks within the DA-CVAE’s encoder, namely, E1 and E2. The first sub-network, the content encoder E1, is designed to process a real anomaly sample $x\in R^{D}$, where *D* signifies the dimensionality of the feature space. The purpose of the content encoder E1 is to learn a mapping that extracts unique, class-agnostic content features, $f_{1}=E_{1}\left(x\right)$, from a specific sample. In doing so, E1 effectively parameterizes the content feature distribution, p(z|x), which represents the sample in the latent space. The condition encoder E2 takes the corresponding attack category label, denoted as $y\in {\left\{0,1\right\}}^{K}$, as its conditional input. This label is one-hot encoded, and *K* represents the total number of attack classes. The purpose of the condition encoder E2 is to learn a mapping to the class-conditional prior distribution, p(z|y). This distribution serves as a global prototype for the given category, and its parameterizing feature vector is given by $f_{2}=E_{2}\left(y\right)$. The class prototypes used by DA-CVAE in the data augmentation process can be regarded as being formed by the parameters of the class-conditional prior distribution. The parameters here are trainable Gaussian parameters in the latent space, which are mainly used to constrain the latent variables of each category sample to cluster near the corresponding category prototype. It should be noted that the category prototype is not a point but a probability distribution, and the distance between each sample and its corresponding category prototype is calculated by KL divergence.

The two encoders, E1 and E2, output feature vectors $f_{1}$ and $f_{2}$, which, in turn, parameterize two separate probability distributions. A primary training objective of the model is to align the content feature distribution p(z|x), derived from a specific sample, with the class prototypical distribution p(z|y), derived from its corresponding category. This alignment is enforced by minimizing the Kullback–Leibler (KL) Divergence between the two distributions.

In the training phase, the latent variable *z* is sampled from the conditional posterior distribution p(z|x,y), which is parameterized by the joint outputs of the content encoder E1 and the class encoder E2. For the decoding stage, the sampled content feature $f_{1}$ (representing sample-specific information) is concatenated with the class feature $f_{2}$ (representing the generic style of the category). This fusion of features ensures that the reconstruction process is conditioned on both aspects. The resulting concatenated vector $\left[f_{1},f_{2}\right]$ is then fed into the decoder, which guarantees that the generation process remains conditioned on the target class information. The decoder network’s function is to map the latent vector back into the original data space, ultimately yielding a generated anomaly sample, $x^{\prime }$. This synthetic sample shares the same class label as the input *x* while exhibiting novel feature characteristics.

The training loss function for the DA-CVAE consists of a weighted sum of two components: a reconstruction loss, which quantifies the discrepancy between the original input *x* and the reconstructed output $x^{\prime }$; and the previously mentioned KL divergence regularization term.

Following the training phase, the DA-CVAE is utilized to generate high-quality synthetic data for specified minority attack classes. The generation process involves sampling a latent vector *z* from the conditional posterior distribution p(z|x,y). This distribution is jointly defined by the content encoder E1 (which processes the input sample *x*) and the category encoder E2 (which processes the class condition *y*). The sampled vector *z* is then passed to the decoder to synthesize a new data instance, thereby creating a more class-balanced training set for the subsequent classification model.

Since DA-CVAE relies on the richness of data samples during training, if the original data of minority-class samples themselves are biased, then the synthetic data will further amplify this bias, which may have a negative impact on the classification results. Second, if the difference between two minority-class samples is small, the synthetic data may further confuse the classification boundary between the two. Therefore, we have set reasonable limits on the volume of synthetic data.

### 3.3. Cost-Sensitive CVAE for Multi-Class Classification

Subsequent to the data balancing phase performed by the DA-CVAE, we introduce our novel Cost-Sensitive Multi-Class Classification Conditional Variational Autoencoder (CSMC-CVAE), which is designed for precise attack type identification. Although conceptually rooted in generative models, the proposed model is fundamentally discriminative in its objective, aiming for classification rather than data generation. As illustrated in the lower part of Figure 1, the CSMC-CVAE utilizes a conditionally symmetric architecture to reconceptualize the classification problem. Specifically, it transforms the task into a problem of evaluating the likelihood of a candidate class given the evidence of a sample, which is essentially a probabilistic matching problem.

Similar to the DA-CVAE, the CSMC-CVAE utilizes a parallel dual-encoder structure, but with a crucial difference: the input and condition are symmetrically swapped. Furthermore, its decoder is substituted with a specialized classification head. The encoder architecture of the CSMC-CVAE is composed of two parallel sub-networks, hereafter referred to as $E_{1}^{\prime }$ and $E_{2}^{\prime }$. The first sub-network, the category prototype encoder E’1, receives a candidate attack category label, denoted as $y\in {\left\{0,1\right\}}^{K}$, as its conditional input. The label *y* is a one-hot vector representation, with *K* representing the total number of distinct attack classes. The $E_{1}^{\prime }$ sub-network is tasked with learning a mapping from the category label *y* to its corresponding prototypical distribution p(z|y) in the latent space. This process yields a feature representation $f_{1}^{\prime }$, which is formally defined as $f_{1}^{\prime }=E_{1}^{\prime }\left(y\right)$. The sample feature encoder, $E_{2}^{\prime }$, accepts a real anomaly sample $x\in R^{D}$ as input, where D is the feature dimension. The role of the $E_{2}^{\prime }$ encoder is to learn a mapping from the input sample *x* to its content feature distribution p(z|x). The output of this encoder is a feature representation, $f_{2}^{\prime }=E_{2}^{\prime }\left(x\right)$.

A core objective of the model’s training is the minimization of the KL divergence between these two distributions. This process ensures that samples belonging to the same category learn to conform to a shared distributional pattern within the latent space, a pattern that is dictated by the corresponding class label. The classification of a test sample $x_{test}$ during the inference stage is performed by identifying the attack category $\hat{y}$ that minimizes the KL divergence between the sample’s content distribution and the category’s prototypical distribution. This process is formally expressed as follows:

(1) $$\hat{y}=\arg\min_{y_{i}\in Y}D_{KL}(q\left(z|x_{test})||p(z|y_{i})\right)$$

The sampled category and content features are then concatenated to form a composite feature vector, which serves as the input to the final classification head. The responsibility of this classification head is to infer and output the definitive class label for the given anomaly sample.

Standard classification models conventionally rely on the minimization of an average loss function, like cross-entropy, for their training. This approach, however, has a significant shortcoming: the loss function becomes dominated by the prevalent majority classes within the training data. Consequently, the model’s ability to effectively learn and recognize samples from underrepresented minority classes is compromised. Despite the data-level rebalancing achieved by the DA-CVAE, the efficacy of data augmentation may remain limited for certain minority classes. Specifically, for classes with high feature complexity, generating high-fidelity synthetic samples is non-trivial. Consequently, we introduce cost-sensitive learning as an algorithm-level solution to further intensify the model’s focus on these underrepresented categories.

The fundamental concept of cost-sensitive learning is to assign differential misclassification costs to various classes. This is operationalized by weighting the loss function according to these costs, thereby guiding the model’s optimization process to place greater emphasis on the accurate prediction of classes associated with higher penalties. For each class $y_{i}$, a corresponding cost weight $C_{y_{i}}$ is assigned. This cost weight is generally set to be inversely proportional to the sample size of the corresponding class. In other words, classes with fewer samples are assigned a higher cost weight. For instance, the weights can be formulated as follows:

(2) $$C_{y_{i}}=\gamma \frac{N_{total}}{N_{y_{i}}}$$

where $N_{total}$ is the total number of training samples, $N_{y_{i}}$ is the number of samples in class $y_{i}$, and γ is a hyperparameter. This strategy effectively compels the model during training to prioritize the correction of errors on minority classes, since the contribution of these misclassified samples to the overall loss is magnified by their cost weights. We use a strategy based on the inverse frequency of categories to automatically generate costs ($C_{y_{i}}$). This approach has the following benefits:

1. Our approach automates cost assignment by using the inverse of class frequencies. This method obviates the need for subjective, manual cost matrix definition by domain experts, ensuring that the weighting scheme is transparent and highly reproducible.
2. By assigning higher weights to minority classes, the contribution of their misclassification errors to the total loss is significantly magnified. This compels the optimization process to prioritize learning from these underrepresented samples, directly counteracting the learning bias induced by the dominant majority classes.
3. The frequency-inverse weighting scheme is inherently adaptive and does not require prior domain knowledge of attack severity. Costs are automatically inferred from the data distribution, allowing our framework to be readily applied to diverse datasets without manual recalibration, which enhances its versatility and practical applicability.

During its training, the total loss function for the CSMC-CVAE, $L_{joint}$, consists of two parts:

(3) $$L_{joint}=\alpha L_{KL}+\beta L_{CSL}$$

in which $L_{KL}$ denotes the Kullback–Leibler (KL) divergence between the category prototype distribution p(z|y) and the sample content distribution p(z|x). The coefficients α and β are tunable hyperparameters that serve to balance the latent space’s distributional consistency against the multi-class classification objective. $L_{CSL}$ represents our proposed cost-sensitive loss, which is formulated as follows:

(4) $$L_{CSL}=\sum_{i=1}^{N}C_{y_{i}}\cdot L\left(\hat{y_{i}},y_{i}\right)$$

where $y_{i}$ is the true class label of the *i*-th sample, $\hat{y_{i}}$ is the model’s predicted output for the *i*-th sample, and $C_{y_{i}}$ is the cost weight corresponding to the true class $y_{i}$. The formulation above implies that the base loss for each instance, $L(\hat{y_{i}},y_{i})$, is modulated by the cost weight $C_{y_{i}}$ associated with its true class $y_{i}$.

The optimization of the joint loss function $L_{joint}$ enables the CSMC-CVAE to do more than just learn a classification task. Crucially, it directs the model to prioritize and bolster its predictive capabilities for underrepresented attack classes. Consequently, the model’s ultimate output, the classification of an anomaly sample, is characterized not only by high overall accuracy but, more importantly, by a markedly superior capability of identifying attack types that are rare yet potentially of high impact.

## 4. Experiments and Result Analysis

In this section, we systematically evaluate and validate the effectiveness of our proposed CSCVAE-NID framework through a comprehensive suite of experiments. We begin by detailing the experimental setup, which encompasses the dataset descriptions, the chosen performance evaluation metrics, and the key hyperparameter settings for our models. Next, we present the performance of the CSCVAE-NID framework on the multi-class intrusion detection task. A comprehensive comparison against various baseline and state-of-the-art methods is then conducted to fully assess its superiority. Furthermore, we delve into how the model’s performance is affected by different levels of class imbalance. Lastly, in order to ascertain the individual contribution of each innovative component within the framework, a series of thorough ablation studies were designed and conducted.

### 4.1. Experimental Setup

**Datasets:** The effectiveness of the proposed framework was comprehensively validated using two well-established benchmark datasets in the domain of network anomaly detection: CICIDS-2017 [11] and UNSW-NB15 [16,17,18]. The CICIDS-2017 dataset encompasses network traffic data spanning a continuous five-day period. The dataset is structured such that the traffic on the first day is purely benign. The subsequent four days, however, are populated with a variety of contemporary network attacks, which encompass brute-force FTP, brute-force SSH, Denial of Service (DoS), Heartbleed, web attacks, infiltration, botnet, and Distributed Denial of Service (DDoS). Every instance within this dataset comprises 78 network traffic features and a single class label, encompassing both the normal category and various common attack types. The UNSW-NB15 dataset, in its original form, comprises nine distinct attack categories. Each data instance is characterized by a 47-dimensional feature vector and a corresponding class label. For this experiment, we selected five representative categories for investigation: one normal class and four distinct attack classes. These four attack categories were chosen to encompass both prevalent and rare sample types. Specifically, DoS and reconnaissance represent high-frequency attacks abundant in the dataset, whereas Shellcode and Worms represent low-frequency attacks with very limited samples. The inclusion of the latter is intended to evaluate the model’s performance under conditions of extreme class imbalance. Table 1 provides the detailed statistical information for the training data utilized in our experiments.

Given the substantial variance in scales and numerical ranges across the feature attributes of the dataset, a normalization step was applied. All feature values were linearly scaled to the closed interval of [0, 1]. This transformation is formally defined by the mathematical equation shown below:

(5) $$x^{*}=\frac{x-x_{min}}{x_{max}-x_{min}}$$

where *x* and $x^{*}$ represent the original data and the normalized data, respectively, while $x_{min}$ and $x_{max}$ denote the minimum and maximum values in the current attribute, respectively.

**Evaluation Metrics:** To accurately assess the efficacy of our proposed method, we employed three key evaluation metrics: Precision, Recall, and F1-score. For simplicity, let $N_{TP}$ be the number of anomaly samples classified as attacks, $N_{TN}$ be the number of normal samples classified as normal, $N_{FP}$ be the number of normal samples classified as attacks, and $N_{FN}$ be the number of anomaly samples classified as normal. The metrics are then defined as follows:

(1) Precision: Precision measures the proportion of true positive instances among all instances classified as positive (anomalous), and $\mathrm{Precision}=\frac{N_{TP}}{N_{TP}+N_{FP}}$.
(2) Recall: Recall is the percentage of correctly predicted anomaly samples out of the total number of actual anomaly samples, and $\mathrm{Recall}=\frac{N_{TP}}{N_{TP}+N_{FN}}$.
(3) F1-Score: The F1-score is the harmonic mean of Precision and Recall, providing a single metric to measure the overall detection accuracy of a model, and F1-$\mathrm{score}\frac{2\times (\mathrm{Precision}\times \mathrm{Recall})}{\mathrm{Precision}+\mathrm{Recall}}$.

Table 2 presents the results for the three evaluation metrics, which are derived from the confusion matrix.

Our proposed method was implemented and evaluated on an NVIDIA A30 GPU with the PyTorch [46] framework. Specifically, the DA-CVAE for data augmentation and the CSMC-CVAE for multi-class classification share a nearly identical encoder–decoder architecture, the specifics of which are detailed in Table 3. It is important to note that a crucial modification was made for the latter model, the CSMC-CVAE. Specifically, its final deconvolutional layer (DeConv4) was replaced by a specialized classification head to facilitate the multi-class prediction task. We employed the Adam [47] optimizer for the two-stage training of the DA-CVAE and the CSMC-CVAE. The initial learning rates were set to 0.0005 and 0.0002, respectively, followed by a gradual decay based on the cosine annealing schedule [48]. Acknowledging the disparities in scale and complexity between the UNSW-NB15 and CICIDS2017 datasets, the models were trained for 50 and 60 epochs, respectively, with a uniform batch size of 128. During the specific training process, our objective was to achieve the highest possible macro F1-score. To this end, we used a small-batch validation set to empirically fine-tune the hyperparameters α, β, and γ. The hyperparameters were configured as follows: γ in Equation (2) was set to 0.2, and the coefficients α and β in Equation (3) were assigned values of 0.25 and 0.75, respectively.

As shown in Table 4, our method exhibits competitive training efficiency, being faster than TMG-IDS, SALAD, and DCHAE. While its total training time is 0.39 h longer than that of CAEP, this is offset by its superior average inference speed, which significantly surpasses that of all compared state-of-the-art methods.

### 4.2. Experimental Results

This section presents an extensive set of validation experiments conducted on the CICIDS2017 and UNSW-NB15 datasets, encompassing both binary and multi-class classification scenarios.

#### 4.2.1. Comparisons with State-of-the-Art Methods

**Binary Classification Results:** To initially assess the efficacy of our framework for the foundational task of anomaly detection, we performed binary classification experiments on the preprocessed UNSW-NB15 and CICIDS-2017 datasets. In this set of experiments, a binary classification scheme was adopted by aggregating all disparate attack traffic types into a unified “Attack” class, in contrast to the “Normal” class. Table 5 details the Precision, Recall, and F1-score achieved by each model for both the normal and attack classes. The final row, labeled “Macro-”, corresponds to the macro-average of the three evaluation metrics. The macro-average was derived by calculating the simple arithmetic mean of the per-class metric scores, thus providing an impartial evaluation of the model’s aggregate performance on the imbalanced dataset. The experimental results unequivocally demonstrate that the proposed CSCVAE-NID framework outperforms all competing methods on both datasets. Table 5 presents the experimental results for the binary classification task on the UNSW-NB15 dataset. A clear observation from the results is that models built upon deep learning principles or featuring tailored architectures, such as DCHAE and CAEP, consistently outperform their counterparts. The DCHAE model stands out as the best-performing baseline among all comparative methods, attaining macro-averaged Precision, Recall, and F1-scores of circa 0.971. Building upon this strong baseline, our proposed CSCVAE-NID framework demonstrates a notable further enhancement in performance. Focusing on the macro-averaged metrics, our proposed method demonstrates state-of-the-art performance, achieving optimal scores of 0.988 for Precision, 0.984 for Recall, and 0.986 for F1-score. Our model outperforms the strongest baseline, CAEP, with relative improvements of 1.7%, 2.0%, and 1.9% in these three key metrics, respectively. In conclusion, the results from the binary classification experiments unequivocally validate the efficacy of our proposed method. It not only demonstrates a superior ability to differentiate between normal and anomalous traffic but has also established a new benchmark for state-of-the-art detection performance.

In Table 6, for the CICIDS2017 dataset, the CSCVAE-NID framework demonstrated superior performance, with macro-averaged Precision, Recall, and F1-scores reaching 0.978, 0.985, and 0.981, respectively. These results represent a significant improvement over all other methods under comparison. Specifically, our CSCVAE-NID outperforms the best-performing baseline, DCHAE, by 0.7%, 1.3%, and 1.0% in terms of macro-averaged Precision, Recall, and F1-score, respectively.

**Multi-Class Classification Results:**  Table 7 presents the experimental results for the multi-class classification task on the UNSW-NB15 dataset. This dataset presents a significant challenge to classification models due to the presence of rare attack categories, such as “Shellcode” and “Worms”, which are characterized by an extreme scarcity of samples. An examination of the table reveals that the SALAD model exhibits the most competitive performance among all baseline methods in terms of macro-averaged metrics. It attained scores of 0.908 for Precision, 0.914 for Recall, and 0.911 for F1-score. Surpassing this leading baseline, our proposed CSCVAE-NID framework achieves a new level of performance. Focusing on the macro-averaged metrics, our proposed method demonstrates the best performance, achieving optimal scores of 0.934 for Precision, 0.933 for Recall, and 0.933 for F1-score. Our model outperforms the strongest baseline, SALAD, with relative improvements of 2.86%, 2.08%, and 2.41% in these three key metrics, respectively. This outcome underscores the robustness of our model. Even when confronted with the complexity and severe class imbalance of the UNSW-NB15 dataset, our framework demonstrates the ability to accurately classify not only normal traffic but also diverse attack types, thereby establishing its superior and optimal detection efficacy.

Table 8, in addition, presents the performance of the models on the multi-class classification task using the CICIDS-2017 dataset. An analysis of the baseline models’ performance on this dataset also reveals a significant challenge in accurately classifying attack categories that are underrepresented. To illustrate, the RFFE model exhibited a notably low Recall for the underrepresented “Web Attack” (label 4) and “Bot” (label 5) classes, with scores of only 0.689 and 0.577, respectively. Even though advanced approaches like MF-Net and TMG-IDS have bolstered the detection of majority-class samples, accurately identifying these rare attack categories continues to be a significant challenge for them. In the multi-class classification task on this dataset, TMG-IDS emerged as the top-performing model among all baselines, achieving macro-averaged Precision, Recall, and F1-scores of 0.949, 0.953, and 0.951, respectively. Once again, our proposed CSCVAE-NID framework surpasses this strong baseline by further boosting the three key macro-averaged metrics to 0.977, 0.982, and 0.980, respectively. This constitutes a significant enhancement of 2.95%, 3.04%, and 3.05%, respectively, over the leading baseline model, TMG-IDS.

#### 4.2.2. Ablation Studies

Effectiveness of Key Components: Ablation studies were conducted to verify the effectiveness of each enhancement made to our model for the multi-class classification task. Table 9 presents the detection results obtained from various combinations of our proposed enhancements. The first combination, denoted as (1), constitutes the baseline model, which excludes all of our proposed enhancements. Specifically, this baseline consists of a standard multi-class CVAE (MC-CVAE) trained exclusively on the original, imbalanced data, without the application of any cost-sensitive learning strategy. Under this setup, the misclassification costs are treated as uniform across all classes (i.e., $C_{y_{i}}=1$ for all *i*). Adding the DA-CVAE data augmentation module in Combination (2) yielded a substantial boost in the overall performance of the model. On the UNSW-NB15 dataset, Precision, Recall, and F1-score were boosted by approximately 3.7%, 1.9%, and 2.8%, respectively, while on the CICIDS2017 dataset, the corresponding improvements were about 2.3%, 1.8%, and 2.0%, respectively. Combination (3) introduces the cost-sensitive learning strategy to the baseline model without data augmentation. This allows us to isolate the impact of the algorithm-level enhancement. On the UNSW-NB15 dataset, this addition elevates the Precision, Recall, and F1-score to 0.968, 0.970, and 0.969, respectively, outperforming the data-level augmentation of Combination (2). A similar trend is observed on the CICIDS2017 dataset. The incremental contribution of integrating both strategies is evident in Combination (4), which represents our full CSCVAE-NID framework. When comparing Combination (4) to Combination (2) (which only has data augmentation), the further integration of the cost-sensitive strategy boosts the F1-score by an additional 3.2% on the UNSW-NB15 dataset (from 0.948 to 0.980) and by 2.1% on the CICIDS2017 dataset (from 0.912 to 0.933). Combination (4), which incorporates all of our proposed enhancements, achieves the final optimal results. The ablation study results confirm that the incorporation of each component into our methodology progressively enhances the final detection performance. This validates that each modification and enhancement within the CSCVAE-NID framework is indeed meaningful and contributes to the overall efficacy.

To investigate the impact of key hyperparameters on model performance, we performed a sensitivity analysis focusing on the crucial hyperparameter γ. Our investigation specifically focused on the hyperparameter γ, a crucial component of the CSMC-CVAE’s joint loss function. The role of γ is to control the trade-off between the cost-sensitive classification loss and the KL divergence regularization term, and its impact was thoroughly analyzed through a series of experiments. Table 10 illustrates the effects of varying the hyperparameter γ on the multi-class detection performance across the UNSW-NB15 and CICIDS-2017 datasets.

A clear observation from the results in Table 10 is that the model’s performance, as measured by Precision, Recall, and F1-score, is highly sensitive to the choice of the hyperparameter γ. The model’s performance metrics, particularly the F1-score, peak when the hyperparameter γ is set to approximately 0.20. At the optimal setting of γ = 0.20, the F1-score achieved was 0.980 on the UNSW-NB15 dataset. For the CICIDS-2017 dataset, this same setting yielded the peak F1-score of 0.933. This finding underscores the robustness of our proposed model across diverse datasets. Furthermore, it indicates that the optimal trade-off point for its key hyperparameters is largely consistent, irrespective of the dataset. A decrease in the model’s baseline performance is observed when the value of γ is suboptimal on the lower side. This phenomenon can be attributed to the reduced emphasis on the KL divergence term during optimization. A smaller γ provides insufficient regularization for the latent space, leading to unstable learning of the category prototypes and, ultimately, a degradation in classification accuracy. Conversely, an overly large value for γ causes the model to over-prioritize the classification of minority classes. This comes at the cost of neglecting the detection of majority-class samples, resulting in a decline in the macro-averaged performance metrics.

Our key innovation for handling imbalanced data is the DA-CVAE. We benchmarked its performance against three state-of-the-art oversampling techniques [49,50,51] using the CICIDS-2017 dataset. In this comparative study, the synthetic data generated by each method, including our DA-CVAE, was used to train the same CSMC-CVAE classifier. The resulting performance metrics are summarized in Table 11.

On aggregate, the experimental findings confirm that an optimal equilibrium exists between the cost-sensitive classification objective and the latent space distribution regularization in our CSCVAE-NID framework. The hyperparameter γ serves as the critical control parameter for modulating this equilibrium. A well-chosen value for the hyperparameter γ ensures a synergistic interplay between the cost-sensitive learning strategy and the CVAE’s generative classification principle. This synergy is crucial for attaining the optimal detection efficacy.

## 5. Conclusions

In this work, we present the CSCVAE-NID, a novel two-stage framework for network intrusion detection. This framework is specifically engineered to achieve a significant enhancement in detection performance for a diverse range of attack types, particularly within the challenging context of imbalanced datasets.

To begin with, we address the challenge of underrepresented minority attack classes in the training data by proposing the DA-CVAE (Data Augmentation Conditional Variational Autoencoder). This generative model is engineered to effectively learn the distinct data distribution of each attack category, enabling the conditional generation of high-quality and diverse synthetic samples. By generating synthetic data, the DA-CVAE constructs a more class-balanced training corpus for the downstream classification model. This data-level enhancement provides a solid groundwork for mitigating the challenges posed by the imbalanced class distribution.

Secondly, we introduce the core of our framework: the CSMC-CVAE (Cost-Sensitive Multi-Class Classification Conditional Variational Autoencoder). This model reconceptualizes the classification problem as an inference process centered on probabilistic distribution matching. This principle is operationalized through a novel, conditionally symmetric dual-encoder architecture. Crucially, a cost-sensitive learning strategy is incorporated into the training of the CSMC-CVAE. The introduction of a predefined cost matrix fundamentally alters the model’s optimization objective during training. Instead of solely pursuing the maximization of classification accuracy, the model is guided to minimize the aggregate weighted risk associated with various types of misclassification errors.

Experimental evaluation on the public and challenging CICIDS-2017 dataset reveals that the proposed CSCVAE-NID framework significantly outperforms state-of-the-art approaches in both binary and multi-class classification scenarios.

It is noteworthy that our proposed CSCVAE-NID framework offers a solution to the dual challenges of class imbalance and risk-aware classification, tackling them concurrently at both the data and algorithmic levels. Notably, the DA-CVAE component is designed as a modular and independent data augmentation unit. The high-quality synthetic data generated by this module can serve as an input for any sophisticated intrusion detection methodology, highlighting its excellent extensibility.

However, it is also important to discuss the inherent limitations of this data-level approach. Specifically, the effectiveness of the data augmentation provided by the DA-CVAE in the first stage is of importance to the overall performance of our framework. When the processed dataset exhibits severe class imbalance, i.e., the sample size of some classes is extremely small, the DA-CVAE has difficulty in effectively augmenting such data. This represents a challenge inherent to most existing data augmentation techniques. It is our hope that acknowledging this limitation will stimulate further targeted research and exploration in this area by the academic community.

## Figures and Tables

**Table 1:** Detailed statistics of the training datasets before and after augmentation.

Dataset	Label	Category	Original Training Set	Augmented Training Set
UNSW-NB15	0	Normal	56,000	56,000
	1	DoS	12,264	22,264
	2	Reconnaissance	10,491	20,491
	3	Shellcode	1133	2133
	4	Worms	130	230
CICIDS2017	0	Benign	105,222	105,222
	1	DoS	21,550	31,550
	2	Port Scan	10,809	20,809
	3	Brute Force	5235	7235
	4	Web Attack	1476	2476
	5	Bot	857	1057

**Table 2:** Structure of the confusion matrix.

		Predicted Class	
		Attack	Normal
**Actual Class**	**Attack**	True Positive (TP)	False Negative (FN)
	**Normal**	False Positive (FP)	True Negative (TN)

**Table 3:** The encoder–decoder architecture of the model.

Component	Layer	Filter Size	Stride
Encoder	Conv1	3 × 3	(2, 2)
	Conv2		(1, 1)
	Conv3		(2, 2)
	Conv4		(1, 1)
Decoder	DeConv1		(1, 1)
	DeConv2		(2, 2)
	DeConv3		(1, 1)
	DeConv4		(2, 2)
Multi-Classification Header	Dense(128)		
	Softmax		

**Table 4:** Comparison of model time efficiency on the CICIDS-2017 dataset.

Method	Dataset	Total Training Time (h)	Average Inference Time (ms)
TMG-IDS	CICIDS-2017	8.41	4.64
SALAD		8.35	1.23
DCHAE		8.29	2.58
CAEP		7.83	3.71
**CSCVAE-NID (Ours)**		**8.22**	**0.90**

**Table 5:** Binary classification results (UNSW-NB15).

**Label**	**RFFE**			**IDS-INT**		
	Precision	Recall	F1-score	Precision	Recall	F1-score
Normal	0.869	0.882	0.875	0.933	0.927	0.930
Attack	0.857	0.826	0.841	0.930	0.908	0.919
Macro-	0.866	0.867	0.866	0.932	0.922	0.927
**Label**	**MF-Net**			**TMG-IDS**		
	Precision	Recall	F1-score	Precision	Recall	F1-score
Normal	0.925	0.916	0.920	0.967	0.958	0.962
Attack	0.941	0.936	0.938	0.972	0.951	0.961
Macro-	0.929	0.922	0.925	0.970	0.956	0.963
**Label**	**SALAD**			**DCHAE**		
	Precision	Recall	F1-score	Precision	Recall	F1-score
Normal	0.946	0.940	0.943	0.961	0.972	0.966
Attack	0.962	0.935	0.948	0.956	0.968	0.962
Macro-	0.950	0.939	0.944	0.960	0.971	0.965
**Label**	**CAEP**			**CSCVAE-NID (Ours)**		
	Precision	Recall	F1-score	Precision	Recall	F1-score
Normal	0.975	0.968	0.971	**0.989**	**0.981**	**0.985**
Attack	0.961	0.954	0.957	**0.985**	**0.992**	**0.988**
Macro-	0.971	0.964	0.967	**0.988**	**0.984**	**0.986**

**Table 6:** Binary classification results (CICIDS2017).

**Label**	**RFFE**			**IDS-INT**		
	Precision	Recall	F1-score	Precision	Recall	F1-score
Normal	0.893	0.904	0.898	0.925	0.941	0.933
Attack	0.858	0.873	0.865	0.931	0.916	0.923
Macro-	0.883	0.895	0.889	0.927	0.933	0.930
**Label**	**MF-Net**			**TMG-IDS**		
	Precision	Recall	F1-score	Precision	Recall	F1-score
Normal	0.952	0.937	0.944	0.974	0.930	0.951
Attack	0.928	0.940	0.934	0.946	0.953	0.950
Macro-	0.945	0.938	0.941	0.966	0.937	0.951
**Label**	**SALAD**			**DCHAE**		
	Precision	Recall	F1-score	Precision	Recall	F1-score
Normal	0.955	0.941	0.948	0.979	0.975	0.977
Attack	0.936	0.965	0.950	0.952	0.966	0.959
Macro-	0.949	0.948	0.948	0.971	0.972	0.971
**Label**	**CAEP**			**CSCVAE-NID (Ours)**		
	Precision	Recall	F1-score	Precision	Recall	F1-score
Normal	0.964	0.949	0.956	**0.982**	**0.989**	**0.985**
Attack	0.958	0.973	0.965	**0.968**	**0.977**	**0.972**
Macro-	0.962	0.956	0.959	**0.978**	**0.985**	**0.981**

**Table 7:** Multi-class classification results (UNSW-NB15).

**Label**	**RFFE**			**IDS-INT**		
	Precision	Recall	F1-score	Precision	Recall	F1-score
0	0.931	0.879	0.904	0.928	0.901	0.914
1	0.719	0.921	0.808	0.846	0.769	0.806
2	0.545	0.835	0.659	0.653	0.717	0.684
3	0.298	0.352	0.323	0.446	0.570	0.499
4	0.772	0.160	0.265	0.483	0.384	0.428
Macro-	0.838	0.871	0.854	0.871	0.851	0.861
**Label**	**MF-Net**			**TMG-IDS**		
	Precision	Recall	F1-score	Precision	Recall	F1-score
0	0.954	0.909	0.931	0.929	0.951	0.940
1	0.672	0.823	0.740	0.892	0.819	0.854
2	0.559	0.541	0.550	0.821	0.752	0.785
3	0.342	0.578	0.430	0.689	0.550	0.612
4	0.741	0.636	0.684	0.547	0.508	0.527
Macro-	0.850	0.842	0.842	0.905	0.898	0.901
**Label**	**SALAD**			**DCHAE**		
	Precision	Recall	F1-score	Precision	Recall	F1-score
0	0.934	0.944	0.939	0.963	0.920	0.941
1	0.872	0.804	0.837	0.752	0.903	0.820
2	0.859	0.915	0.886	0.671	0.841	0.746
3	0.507	0.701	0.588	0.283	0.707	0.404
4	0.497	0.447	0.471	0.574	0.735	0.645
Macro-	0.908	0.914	0.911	0.882	0.903	0.892
**Label**	**CAEP**			**CSCVAE-NID (Ours)**		
	Precision	Recall	F1-score	Precision	Recall	F1-score
0	0.955	0.946	0.950	**0.974**	**0.959**	**0.966**
1	0.826	0.908	0.865	**0.879**	**0.921**	**0.899**
2	0.771	0.674	0.719	**0.806**	**0.840**	**0.823**
3	0.303	0.512	0.381	**0.768**	**0.693**	**0.729**
4	0.533	0.609	0.568	**0.692**	**0.784**	**0.735**
Macro-	0.901	0.897	0.899	**0.934**	**0.933**	**0.933**

**Table 8:** Multi-class classification results (CICIDS-2017).

**Label**	**RFFE**			**IDS-INT**		
	Precision	Recall	F1-score	Precision	Recall	F1-score
0	0.841	0.874	0.857	0.904	0.918	0.911
1	0.918	0.859	0.888	0.937	0.906	0.921
2	0.902	0.934	0.918	0.926	0.860	0.892
3	0.961	0.782	0.862	0.798	0.851	0.824
4	0.743	0.689	0.715	0.905	0.699	0.789
5	0.892	0.577	0.700	0.931	0.784	0.851
Macro-	0.860	0.868	0.864	0.906	0.906	0.906
**Label**	**MF-Net**			**TMG-IDS**		
	Precision	Recall	F1-score	Precision	Recall	F1-score
0	0.935	0.947	0.941	0.955	0.967	0.961
1	0.972	0.931	0.951	0.926	0.934	0.930
2	0.938	0.984	0.960	0.983	0.925	0.953
3	0.904	0.846	0.874	0.920	0.881	0.900
4	0.896	0.821	0.857	0.874	0.796	0.833
5	0.961	0.919	0.940	0.907	0.942	0.924
Macro-	0.938	0.941	0.939	0.949	0.953	0.951
**Label**	**SALAD**			**DCHAE**		
	Precision	Recall	F1-score	Precision	Recall	F1-score
0	0.929	0.911	0.920	0.946	0.956	0.951
1	0.967	0.963	0.965	0.913	0.943	0.928
2	0.943	0.926	0.934	0.947	0.954	0.950
3	0.950	0.825	0.883	0.932	0.893	0.912
4	0.912	0.907	0.910	0.908	0.849	0.878
5	0.827	0.743	0.783	0.925	0.941	0.933
Macro-	0.935	0.916	0.925	0.939	0.950	0.944
**Label**	**CAEP**			**CSCVAE-NID (Ours)**		
	Precision	Recall	F1-score	Precision	Recall	F1-score
0	0.940	0.981	0.960	**0.979**	**0.983**	**0.981**
1	0.929	0.952	0.940	**0.965**	**0.976**	**0.970**
2	0.951	0.925	0.938	**0.991**	**0.994**	**0.992**
3	0.937	0.956	0.946	**0.983**	**0.978**	**0.980**
4	0.908	0.867	0.887	**1.000**	**1.000**	**1.000**
5	0.944	0.883	0.913	**1.000**	**0.989**	**0.994**
Macro-	0.944	0.938	0.952	**0.977**	**0.982**	**0.980**

**Table 9:** Ablation study results for the multi-class classification task.

Datasets	Metrics	Comb. 1 (MC-CVAE)	Comb. 2 (+DA-CVAE)	Comb. 3 (+Cost-Sens.)	Comb. 4 (CSMC-CVAE)
UNSW-NB15	Precision	0.914	0.951	0.968	**0.977**
	Recall	0.926	0.945	0.970	**0.982**
	F1-score	0.920	0.948	0.969	**0.980**
CICIDS2017	Precision	0.894	0.917	0.928	**0.934**
	Recall	0.890	0.908	0.925	**0.933**
	F1-score	0.892	0.912	0.926	**0.933**

**Table 10:** Impact of the hyperparameter γ on detection performance.

Datasets	UNSW-NB15			CICIDS2017		
	Precision	Recall	F1-Score	Precision	Recall	F1-Score
γ=0.16	0.962	0.965	0.963	0.914	0.912	0.913
γ=0.18	0.973	0.974	0.973	0.926	0.928	0.927
γ=0.20	**0.977**	**0.982**	**0.980**	**0.934**	**0.933**	**0.933**
γ=0.22	0.975	0.971	0.973	0.930	0.924	0.927
γ=0.24	0.967	0.963	0.965	0.923	0.917	0.921
γ=0.26	0.958	0.955	0.956	0.917	0.915	0.916

**Table 11:** Comparison of different data augmentation methods.

Data Augmentation	Dataset	Classifier	Precision	Recall	F1-Score
CTGAN	CICIDS-2017	CSMC-CVAE	0.968	0.971	0.969
BCTGAN			0.971	0.975	0.973
ADASYN			0.959	0.968	0.963
**DA-CVAE (Ours)**			**0.977**	**0.982**	**0.980**

## Data Availability

The data presented in this study are available on request from the corresponding author.
